# Folate-driven changes in snoRNA function: a novel epigenetic-ribosomal axis in hepatocellular carcinoma

**DOI:** 10.1186/s41065-026-00666-x

**Published:** 2026-03-23

**Authors:** Xinxin Song, Deyu Kong, Yongjiao Zi, Xu Wang, Juan Ni

**Affiliations:** 1 College of Life Sciences, Yunnan Normal University, Kunming, 650500 China; 2 Department of Medical Oncology, The First Affiliated Hospital of Kunming Medical University, Kunming, 650000 China; 3 Yeda Institute of Gene and Cell Therapy, Taizhou, 318000 China

**Keywords:** Folate, Hepatocellular carcinoma, Small nucleolar RNA

## Abstract

Folate (FA), essential for DNA synthesis, repair and methylation, is predominantly stored and metabolized in the liver. Dysregulation of folate intake and metabolism is strongly implicated in the pathogenesis of hepatocellular carcinoma (HCC). Although small nucleolar RNAs (snoRNAs) have shown promise as diagnostic and prognostic biomarkers in HCC, the impact of folate deficiency on their expression landscape remains uncharacterized. Here, by performing snoRNA-seq in folate-deficient HepG2 cells, we systematically identified 26 differentially expressed snoRNAs for the first time. Functional enrichment analysis indicated that downregulated snoRNAs were closely associated with cell proliferation pathways. From these, we selected SNORD1B, highly expressed in HCC tissues, correlated with poor prognosis, and significantly suppressed upon 28-day FA deprivation for mechanistic investigation. We demonstrated that FA deficiency coordinately downregulated SNORD1B and its host gene SNHG16 (*P* < 0.05), with strong positive correlation (R^2^ = 0.5104, *P* = 0.0465). This downregulation led to markedly reduced 2′-O-methylation at the G4362 site of 28S rRNA (*P* < 0.01) and impaired ribosomal activity. Expression of snoRNA-binding proteins (FBL, NOP58, NOP56) as well as IL-6 and CDK1 were significantly suppressed (*P* < 0.05), alongside decreased TP53 protein levels (*P* < 0.001). Functional assays confirmed that FA deficiency potently inhibited HepG2 proliferation and migration (*P* < 0.01). Our findings reveal a previously unrecognized mechanism: folate deficiency attenuates SNORD1B-dependent 2′-O-methylation of 28S rRNA, resulting in ribosomal dysfunction and suppression of global protein synthesis, thereby inhibiting HCC cell proliferation and migration. This study provides novel insight into the epigenetic-ribosomal axis of FA-mediated tumor suppression and highlights snoRNAs as potential therapeutic targets in HCC.

**Supplementary Information:**

The online version contains supplementary material available at 10.1186/s41065-026-00666-x.

## Introduction

Hepatocellular carcinoma (HCC), which constitutes 80%–90% of primary liver cancer cases, represents a significant global health challenge [1]. According to 2022 estimates from the International Agency for Research on Cancer (IARC), liver cancer was the sixth most commonly diagnosed cancer worldwide and the third leading cause of cancer-related death, accounting for approximately 760,000 fatalities [2]. Current therapeutic strategies, such as surgical resection, liver transplantation, and chemotherapy, are often ineffective for a majority of patients. This is primarily because the high heterogeneity of HCC and a lack of reliable early biomarkers result in most patients being diagnosed at intermediate or advanced stages, leading to a poor prognosis [3–5].

The liver plays a central role in micronutrient metabolism, and the status of micronutrients is closely linked to the progression of liver disease [6]. Folate (FA), an essential water-soluble B vitamin obtained from the diet, serves as a critical cofactor for DNA synthesis and biomethylation processes. Its metabolically active form, tetrahydrofolate (THF), catalyzes the conversion of dUMP to dTMP, a critical step in DNA synthesis. FA deficiency impairs dTMP biosynthesis, resulting in uracil misincorporation into DNA and genomic instability. Moreover, FA provides one-carbon units for synthesizing S-adenosylmethionine (SAM), the universal methyl donor for epigenetic modifications. Consequently, insufficient FA disrupts SAM production, alters the epigenetic landscape, and dysregulates gene expression [7–9]. Interestingly, the role of FA in cancer appears dualistic: supplementation can improve DNA methylation and mitigate inflammation, yet may paradoxically accelerate precancerous lesions [10]. Conversely, dietary FA restriction may suppress tumor growth by impairing nucleotide synthesis [11–13].

Small nucleolar RNAs (snoRNAs), which range from 60 to 300 nucleotides in length, are predominantly derived from the intronic regions of host genes (SNHGs) [14]. These RNAs primarily direct the post-transcriptional chemical modifications of rRNAs, snRNAs, and tRNAs. Additionally, they participate in a spectrum of other functions, such as transcriptional regulation, alternative polyadenylation, and miRNA-like activities, and have been linked to telomere maintenance, ADP-ribosylation, and chromatin remodeling [15–20]. The dysregulation of snoRNAs has been increasingly linked to oncogenesis, and their presence in stable form in body fluids underscores their utility as non-invasive diagnostic and prognostic biomarkers [21–24]. In HCC, specific snoRNAs have shown promise as molecular markers. Furthermore, emerging evidence suggests that, like other non-coding RNAs, the expression and activity of snoRNAs may be modulated by micronutrient availability [25–27].

However, the effect of folate deficiency on snoRNA expression in HCC has not been elucidated. In this study, HepG2 cells were subjected to folate-sufficient or folate-deficient culture conditions. snoRNA expression profiles were analyzed by RNA sequencing, complemented by data from public databases to evaluate their expression in liver cancer tissues and prognostic value. Functional implications were investigated through experimental validation and pathway enrichment analysis. The results demonstrated that folate deficiency downregulates specific snoRNAs, thereby abrogating their guidance of modifications at target rRNA sites. This disruption led to diminished ribosomal activity and overall protein synthesis capacity, which ultimately inhibited tumor cell proliferation and migration. Our work provides the first evidence that folate deprivation dynamically alters the expression patterns of specific snoRNAs. These findings not only offer new mechanistic insights into the role of folate in HCC but also highlight potential diagnostic and therapeutic targets.

## Materials and methods

### Cell culture

The human hepatocellular carcinoma cell line HepG2 (cat# HB-8065, ATCC) was used in this study. The cells were confirmed to be free of mycoplasma, bacterial, yeast, and fungal contamination. Cells were cultured under two different FA conditions: FA-sufficient and FA-deficient. For the FA-sufficient group, HepG2 cells were maintained in RPMI 1640 medium (GIBCO) containing 2260 nmol/L FA and supplemented with 8% fetal bovine serum (GIBCO, NY, USA), 1% penicillin–streptomycin (GIBCO, NY, USA), and 1% L-glutamine (GIBCO, NY, USA). For the FA-deficient group, cells were cultured in medium with a FA concentration of 22.6 nmol/L.

To prepare the FA-deficient medium, a FA stock solution (500 μg/mL) was first made by dissolving 50 mg FA in 100 mL 0.1 M NaOH under light-protected conditions, followed by filtration through a 0.22 μm filter and stored at −20 °C in the dark. This stock solution was then added to FA-free customized RPMI 1640 base medium to achieve the final concentration of 22.6 nmol/L. The 22.6 nmol/L concentration was selected as inadequate based on our previous studies [28]. Cells were seeded at a density of 3 × 10^5^ cells per 25 cm^2^ culture flask. The culture medium was replaced every 3 days for a total duration of 28 days, during which cells were subcultured every 3–4 days upon reaching 80–90% confluence. At each subculture, cell viability was assessed by trypan blue exclusion assay, and only cultures with viability > 90% were used for subsequent experiments. Cell morphology was monitored daily under an inverted phase-contrast microscope, observing cell attachment, confluence, and any morphological changes such as vacuolization or detachment. All cell cultures were maintained at 37 °C in a humidified incubator with an atmosphere of 5% CO₂.

It should be noted that the fetal bovine serum used contains trace amounts of endogenous FA. However, as the same batch and concentration of FBS was added to both culture conditions, this background contribution does not affect the comparison of biological effects between the 100-fold FA concentration gradient established in this study.

### snoRNA sequencing

snoRNA sequencing was performed by Outstanding Biotech (Shenzhen, China). Total RNA was extracted from HepG2 cells, and three biological replicates were prepared for each experimental condition (FA-sufficient and FA-deficient). RNA integrity was assessed using the Agilent 2100 Bioanalyzer, and all samples used for library construction had an RNA Integrity Number ≥ 7.0, ensuring high-quality RNA for subsequent sequencing.

Library construction was carried out by ligating 3' and 5' adaptors to small RNA ends, followed by cDNA synthesis and PCR amplification to generate double-stranded cDNA libraries. A total of 13 cycles were performed to amplify the library. The libraries were then purified, and fragments with insert sizes of 18–40 bp were selected for sequencing. After construction, library quality was assessed based on insert fragment quantification and effective concentration.

Qualified libraries were pooled in equimolar ratios according to their effective concentrations and sequencing data requirements, and were subsequently sequenced on an Illumina platform with PE50 sequencing strategy. To ensure sufficient coverage for reliable snoRNA expression analysis, we generated an average of 10 million raw reads per sample.

### Data retrieval of snoRNAs from TCGA database

We retrieved snoRNA expression profiles from The Cancer Genome Atlas (TCGA), including data from 369 LIHC (Liver Hepatocellular Carcinoma) tissues and 110 normal tissues. snoRNA expression data were downloaded from the TCGA Data Portal (https://portal.gdc.cancer.gov/). Transcriptome data and gene probe information were obtained, and clinical data files for LIHC patients were also downloaded. The annotation of snoRNAs was based on the GRCh38/hg38 genome assembly with gene annotations from GENCODE.

Data processing was performed using R software (version 4.3.1). Transcriptome data from tumor samples and normal samples were read, gene names were converted, and data were filtered to construct expression matrices. The two datasets were then integrated and normalized. Differential expression analysis between LIHC tissues and normal tissues was performed using the limma R package. The threshold for identifying differentially expressed snoRNAs was set as log2(fold change) ≥ 1 and adjusted P-value < 0.05. The expression levels of differentially expressed snoRNAs were subsequently normalized and log2-transformed for further analysis. Boxplots showing differential expression of snoRNAs between the two groups were generated using the ggpubr R package.

Kaplan–Meier survival analysis was employed to evaluate the relationship between snoRNA expression and overall survival in the LIHC cohort. Gene expression data were combined with clinical survival information, including overall survival and survival status. Patients were stratified into high-expression and low-expression groups based on the median expression value of each snoRNA. Kaplan–Meier survival curves were generated using the survival and survminer R packages, and the log-rank test was used to compare survival differences between groups.

### Gene set enrichment analysis

Enrichment analysis was conducted via the RNAenrich online tool (http://idrblab.cn/rnaenrich/). The list of candidate snoRNAs was submitted to the server with parameters set for the relevant species, RNA type (snoRNA), and ID type. The analysis returned enriched terms across several databases, encompassing biological processes, cellular components, molecular functions, KEGG pathways and diseases, Reactome pathways, SMPDB pathways, TTD diseases, and TTD targets.

### RNA extraction and the quantitative PCR

RNA was isolated with Trizol (Invitrogen, USA) and reverse-transcribed into cDNA using HiScript Q RT SuperMix (+ gDNA wiper; Vazyme, China). All primers (Table S1), designed with Primer Premier 5, were provided by Tsingke Biotechnology Co., Ltd. qPCR assays were conducted with Fast Start Universal SYBR Green Master (GlpBio, USA), in accordance with the manufacturer's protocol. Using GAPDH as an endogenous reference, the relative expression of snoRNAs was determined by the 2^−ΔΔCt^ method.

### RTL-P Analysis of 2′-O-Ribose methylation status

We employed an optimized RTL-P (Reverse Transcription at Low deoxyribonucleoside triphosphate concentrations followed by polymerase chain reaction) approach [29] to analyze 2'-O-ribose methylation levels at specific rRNA sites. This method utilizes site-specific reverse transcription under low dNTP conditions, followed by semi-quantitative PCR amplification. The specific 2'-O-ribose methylation site analyzed in this study was 28S rRNA G4362, and the primer sequences are provided in Additional file 1: Supplementary materials (Table S1).

The specific reverse transcription reaction was performed as follows: 100 ng of RNA, 1 μL of site-specific RT primer, and RNase-free water were combined to a total volume of 6 μL. The mixture was incubated at 70 °C for 10 min, immediately chilled on ice for at least 2 min, and briefly centrifuged. The following reverse transcription reaction solution was then added: 2 μL of 5 × M-MLV buffer, 0.5 μL of dNTPs (low concentration: 1 μM; high concentration: 1 mM), 0.25 μL of RNase inhibitor (40 U/μL), 0.5 μL of RTase M-MLV (RNase H-) (200 U/μL), and RNase-free water to a final volume of 6 μL. The reaction mixture was incubated at 42 °C for 1 h, followed by 70 °C for 15 min, and then cooled on ice. The resulting cDNA was used directly for RT-qPCR amplification.

The methylation level was quantified using the comparative CT method. For each sample, the average CT value of the target gene was normalized to the average CT value of the housekeeping gene to obtain the dCT value. The ddCT value was calculated as the difference between the dCT of the experimental sample and the dCT of the calibrator sample. The relative methylation level was expressed as 2^^−ddCT^. RT efficiency was assessed by comparing the 2^^dCT^ values under low and high dNTP conditions, with higher values indicating lower methylation levels. Statistical analysis was performed to compare methylation levels between the FA-sufficient and FA-deficient groups.

### Ribosomal activity analysis by OP-puro incorporation assay

Ribosome activity was evaluated using an O-propargyl-puromycin (OP-puro) incorporation assay. OP-puro, an analog of puromycin, is incorporated into newly synthesized proteins. OP-puro was added to the culture medium at a final concentration of 50 μM and incubated for 1 h at 37 °C to allow incorporation into nascent polypeptide chains.

After OP-puro incubation, cells were washed twice with PBS and fixed in 0.5 mL of 4% paraformaldehyde on ice for 20 min. Following fixation, cells were washed with PBS and permeabilized in 200 μL of PBS containing 0.1% Triton X-100 and 3% FBS for 20 min at room temperature. The Click-iT reaction solution was prepared as follows: 440 μL of 1 × Click-iT cell reaction buffer, 10 μL of CuSO₄, 50 μL of Click-iT cell buffer additive, and 2.5 μM Alexa Fluor™ 488 azide, brought to a total volume of 500 μL. The reaction solution was used within 15 min of preparation. Each well was incubated with 500 μL of Click-iT reaction solution for 30 min at room temperature in the dark. After incubation, cells were washed twice with PBS and incubated with 200 μL of Hoechst 33,342 for 30 min for nuclear staining.

Fluorescence signals were visualized using a fluorescence microscope with a scale bar of 20 μm. Five randomly selected fields (upper left, lower left, upper right, lower right, and center) were captured per well, with each field containing approximately 15 cells. Fluorescence quantification was performed using ImageJ software with the following parameters: images were converted to 8-bit format, appropriate fluorescence thresholds were set to exclude background interference, and the Analyze Particles function was used to measure the fluorescence area and mean fluorescence intensity per field. Fluorescence intensity correlates with the level of newly synthesized proteins, reflecting ribosome activity. Statistical analysis was performed to compare protein synthesis rates between the FA-sufficient and FA-deficient groups.

### Plate clone formation assay

HepG2 cells were seeded in 6-well plates (Corning, NY, USA) at a density of 1,000 cells per well and cultured for 14 days. The culture medium was replaced every 3–4 days to ensure adequate nutrition. After 14 days, cells were fixed with 4% paraformaldehyde for 30 min and stained with 0.4% crystal violet for 15 min. The cells were gently washed with slow-running water and air-dried. Colonies containing ≥ 50 cells were counted using ImageJ software. All counting was performed in a blinded manner, and each experiment was repeated three times independently.

### 5-Ethynyl-2′-deoxyuridine incorporation

After culturing HepG2 cells under FA-sufficient and FA-deficient conditions for 14 and 28 days, cell proliferation was assessed using the 5-ethynyl-2′-deoxyuridine (EdU) assay kit (Beyotime, China). Cells were seeded in 24-well plates at a density of 3 × 10^4^ cells per well and cultured overnight. EdU was added to the culture medium at a final concentration of 25 μM and incubated for 2 h. After incubation, cells were fixed with 4% paraformaldehyde for 15 min and permeabilized with PBS containing 0.3% Triton X-100 for 10 min. The Click reaction solution was prepared according to the manufacturer's instructions and added to each well for 30 min in the dark. Nuclei were counterstained with DAPI for 8 min. Images were captured using a fluorescence microscope (IX53, Olympus Corporation, Japan) with five randomly selected fields per well (upper left, lower left, upper right, lower right, and center). The percentage of EdU-positive cells was calculated as (number of EdU-positive cells/total number of DAPI-stained nuclei) × 100% using ImageJ software. All image acquisition and analysis were performed in a blinded manner.

### Transwell assay

HepG2 cells were seeded into the upper chambers of Transwell plates (Corning, NY, USA) at a density of 5 × 10^2^ cells per well in 200 μL of serum-free medium. The lower chambers were filled with 800 μL of culture medium containing 20% FBS, with FA concentrations corresponding to the experimental groups (sufficient or deficient). Cells were incubated at 37 °C with 5% CO₂ for 12 h. After incubation, the upper chambers were removed, and non-migrated cells on the upper membrane surface were gently wiped off with a cotton swab moistened with PBS. Cells on the lower membrane surface were fixed with anhydrous ethanol for 30 min at room temperature and stained with 0.4% crystal violet for 20 min. Five randomly selected fields per well were captured under an inverted microscope (IX53, Olympus Corporation, Japan) at 200 × magnification. Migrated cells were counted using ImageJ software. All counting was performed in a blinded manner.

### Statistical analysis

Data are presented as the mean with standard deviation (SD). All statistical analyses were performed using IBM SPSS Statistics 25 (SPSS, Chicago, IL, USA) and graphs were generated with Prism 9.0 (GraphPad, San Diego, CA, USA). For comparisons between two groups, an independent sample t-test was used. For experiments involving multiple groups or multiple time points, one-way or two-way analysis of variance (ANOVA) was performed. Statistical significance was set at *P* < 0.05. Significance levels are indicated as follows: ^*^P < 0.05, ^**^P < 0.01, ^***^P < 0.001.

## Results

### Comparative analysis of the snoRNA expression landscape during folate deficiency

**Fig. 1:**
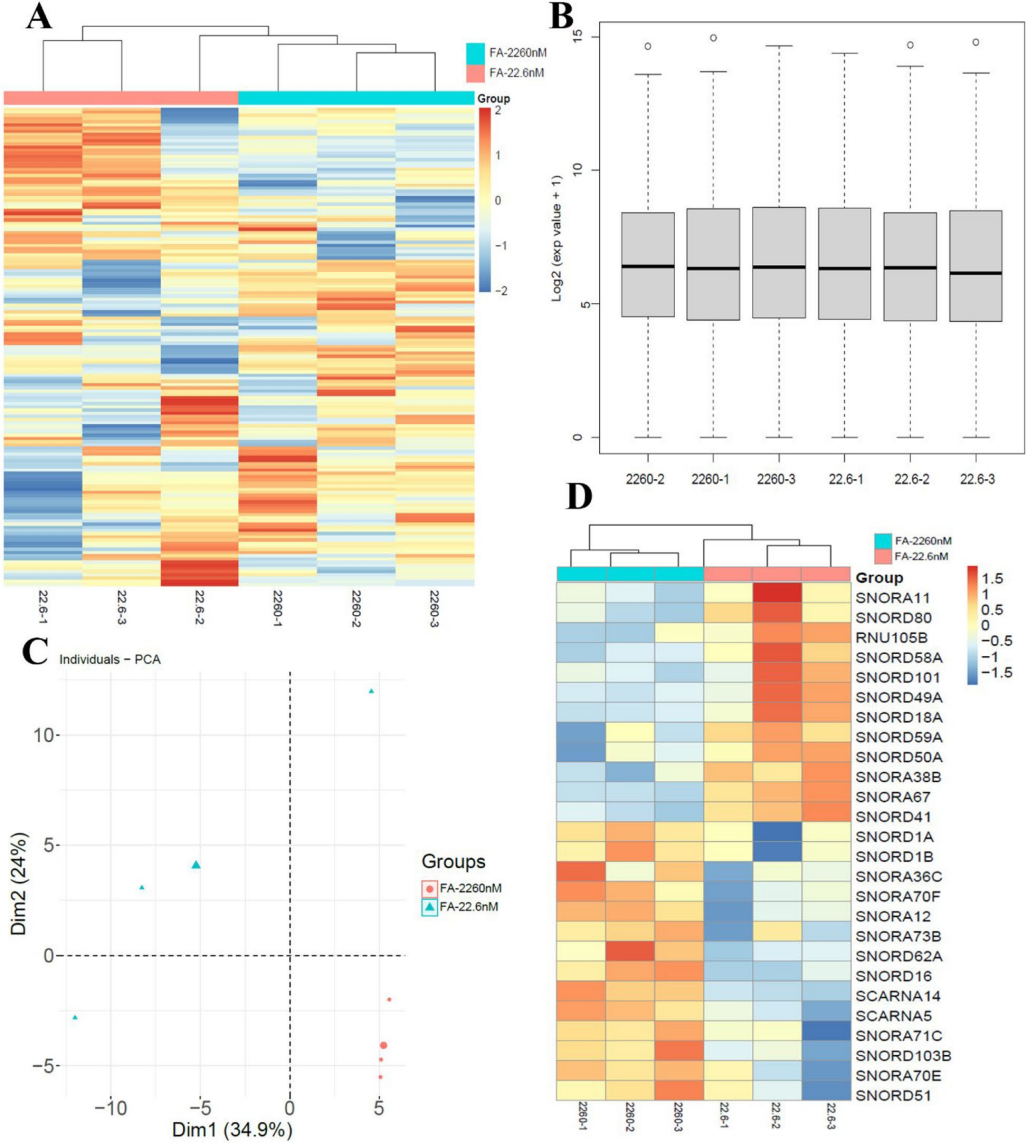
Identification of differentially expressed snoRNAs in HepG2.** A** Heatmap of differentially expressed snoRNAs between groups. **B** Distribution of expression values across the six samples shown by box plot. **C** Principal component analysis of normalized snoRNA expression profiles. Points represent individual samples, larger symbols indicate group means. **D** Cluster analysis of differentially expressed snoRNAs. red: upregulation; blue: downregulation

We profiled snoRNAs in HepG2 employing 28-day cultures under either FA-sufficient or FA-deficient conditions. This analysis identified a total of 151 snoRNAs (Fig. 1A). The box plot demonstrates a consistent data distribution across the six samples and confirms the absence of outliers, indicating robust data quality (Fig. 1B). Principal component analysis (PCA) revealed distinct clustering of samples based on FA status, demonstrating a pronounced influence of FA deficiency on the global snoRNA expression profile (Fig. 1C).

Among them, 12 snoRNAs were significantly up-regulated (including SNORA11、SNORD80、RNU105B、SNORD58A、SNORD101、SNORD49A、SNORD18A、SNORD59A、SNORD50A、SNORD38B、SNORA38B、SNORA67、SNORD41) and 14 snoRNAs were significantly down-regulated (including SNORD1A、SNORD1B、SNORA36C、SNORA70F、SNORA12、SNORA73B、SNORA62A、SNORD16、SCARNA14、SCARNA5、SNORA71C、SNORD103B、SNORA70E、SNORD51) compared with the control group (Fig. 1D).

### Potential biological pathways associated with differentially expressed snoRNAs

**Fig. 2:**
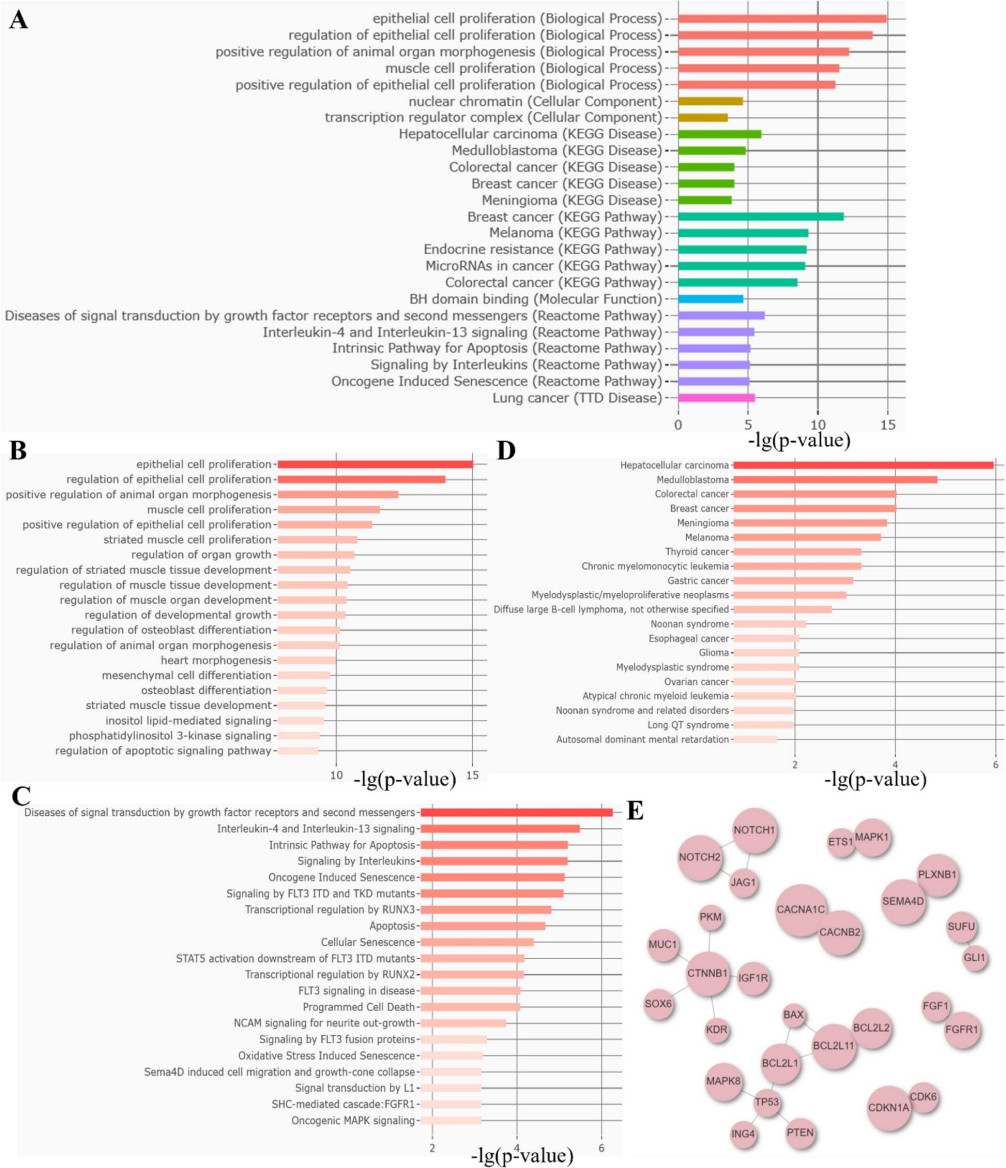
Gene set enrichment analysis of 14 downregulated snoRNAs. **A** Database summary. **B** Biological process enrichment. **C** Molecular function enrichment. **D** KEGG pathway disease enrichment. **E** Protein–protein interaction network. Nodes and edges represent the relationships and the strength of interactions between them

To elucidate the biological pathways linked to the differential snoRNA expression, we conducted Gene Set Enrichment Analysis (GSEA) on the differential snoRNAs using RNAEnrich (http://idrblab.cn/rnaenrich/). This analysis identified multiple enriched gene sets, and the integration of results from multiple databases is summarized (Fig. 2A, S1A). The downregulated snoRNAs were significantly enriched in gene sets governing epithelial cell proliferation, regulation of epithelial cell proliferation, muscle cell proliferation, and positive regulation of animal organ morphogenesis (Fig. 2B). These snoRNAs were significantly enriched in Reactome pathways related to factor receptor signaling, second messenger pathways, and interleukin signaling (Fig. 2C). KEGG disease enrichment analysis identified HCC as the most significantly enriched disease, with additional strong associations for medulloblastoma, colorectal cancer, and breast cancer (Fig. 2D). Furthermore, protein–protein interaction (PPI) network analysis identified TP53, CTNNB1, and BCL2L1 as the primary hub proteins (Fig. 2E).

Upregulated snoRNAs enhance cellular sensing of oxygen, peptides, and chemical stress. Molecular functions were enriched for phosphoprotein, phosphatase, and phosphotyrosine binding. Reactome analysis highlighted non-receptor tyrosine kinase, extranuclear estrogen, and PI3K/AKT signaling in cancer, implicating these snoRNAs in critical growth and metabolic pathways. PPI network analysis identified key hubs, including SMAD4, SMAD2, FOXO1, FOXO4, MYC, SKP2, CDK2, CDK4, and EGFR (Fig. S1).

### The verification results of RT-qPCR were basically consistent

**Fig. 3:**
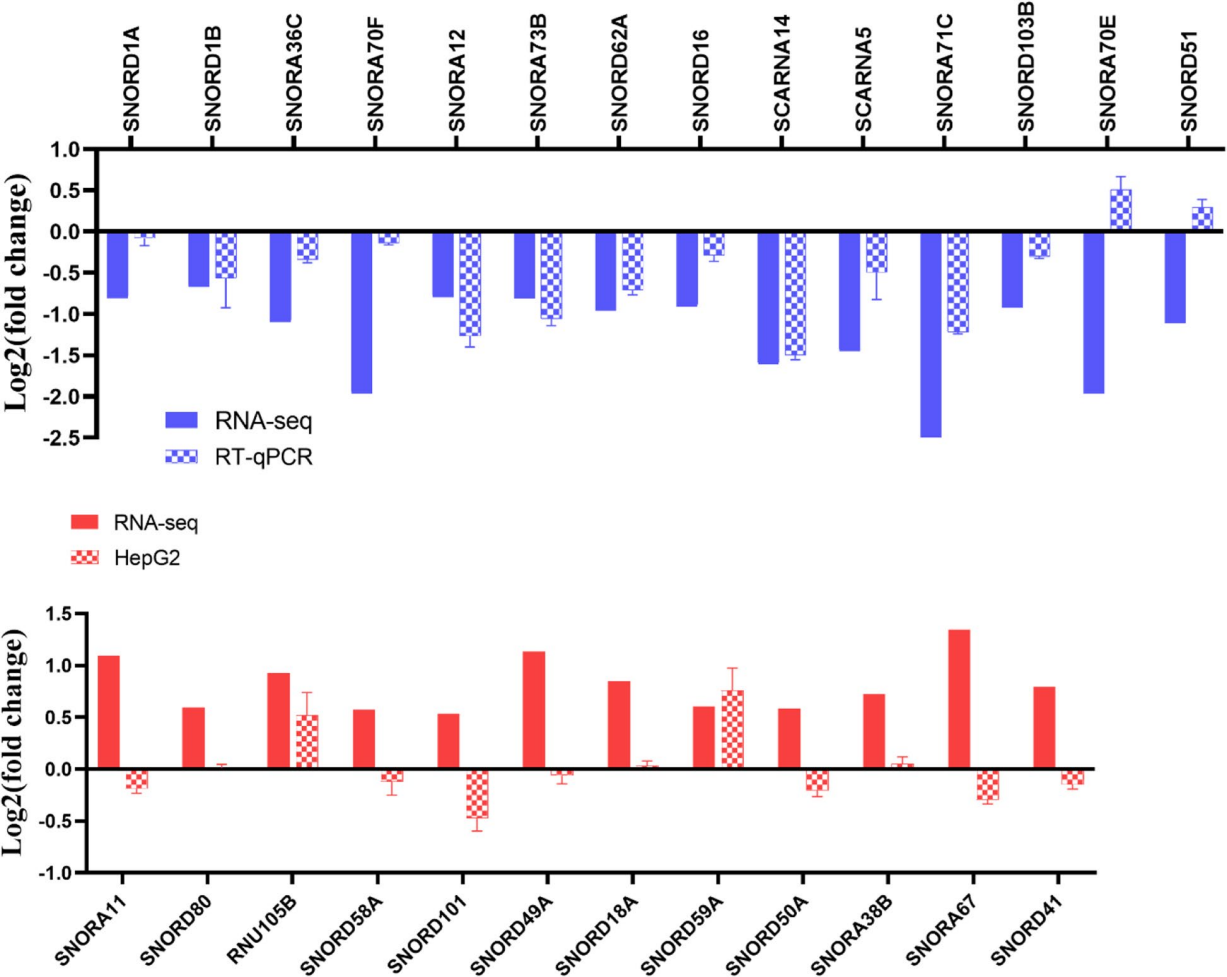
RT-qPCR validation of RNA-Seq results. Comparison of the high-throughput sequencing data and RT-qPCR result of HepG2 in FA deficiency. The bar chart represents the standard error, with the experiment repeated three times

We validated the RNA-seq results for 15 snoRNAs by RT-qPCR across the same RNA samples. The expression patterns measured by both methods were highly consistent (Fig. 3).

### Specific snoRNAs are promising diagnostic and prognostic biomarkers for HCC

**Fig. 4:**
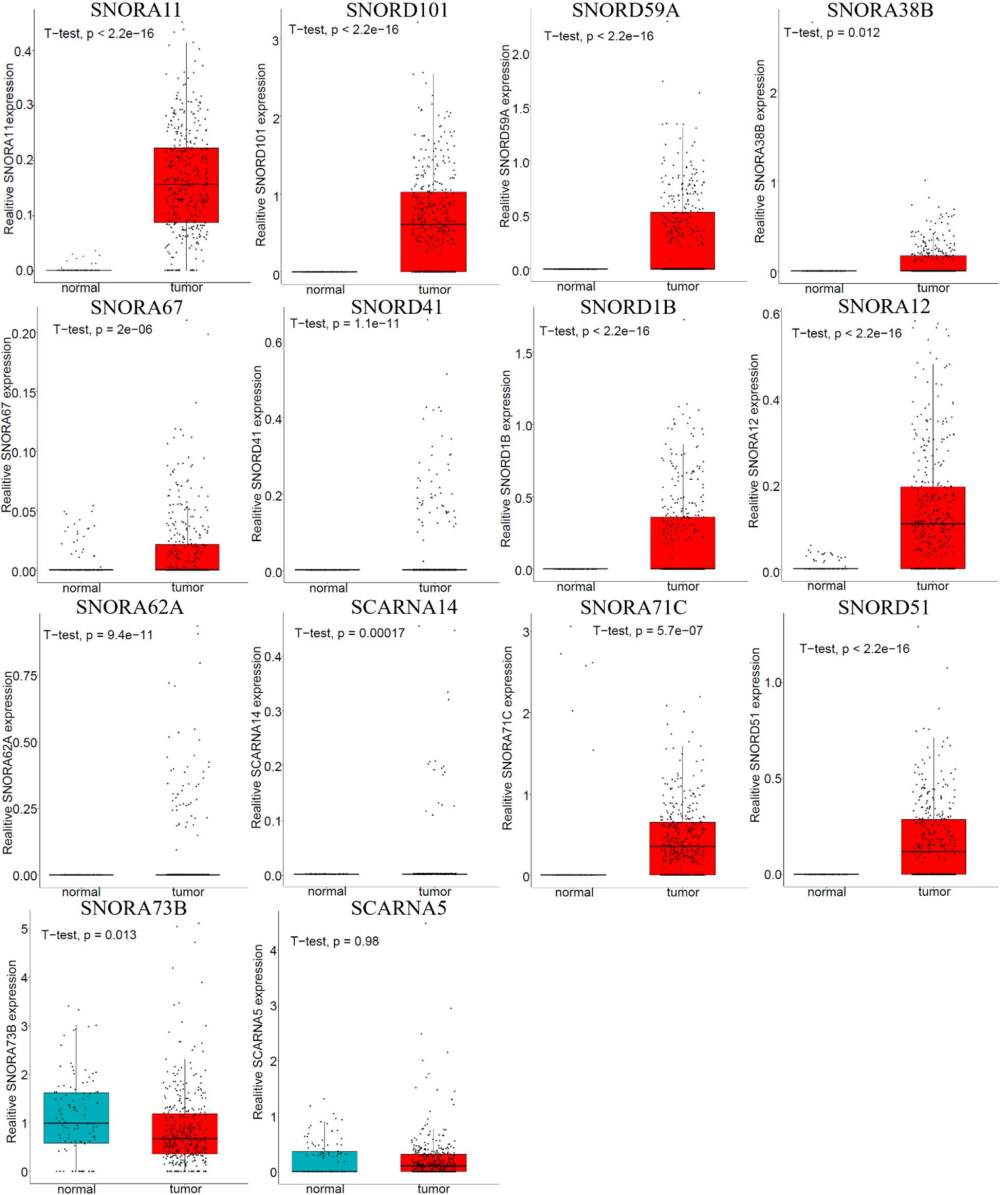
The expression of snoRNA in LIHC tissues and normal liver tissues. Blue pixels indicate normal whereas red indicates tumor

To assess the clinical significance of these snoRNAs, we obtained RNA-seq data for tumor and adjacent normal tissues from TCGA database. Differential expression analysis identified 14 significant snoRNAs. Of these, thirteen (including SNORA11, SNORD101, SNORD59A, SNORA38B, SNORA67, SNORD41, SNORD1B, SNORA70F, SNORA12, SNORA62A, SCARNA14, SNORA71C, SNORD51) were up-regulated in LIHC tissues, while only SNORD73B was significantly down-regulated (Fig. 4). Kaplan–Meier analysis identified high expression of SNORA36C as the sole predictor of poor prognosis among the 15 snoRNAs evaluated in the prognostic signature (Fig. S2).

### Folate availability regulates SNORD1B transcription by acting through its host gene, SNHG16

**Fig. 5:**
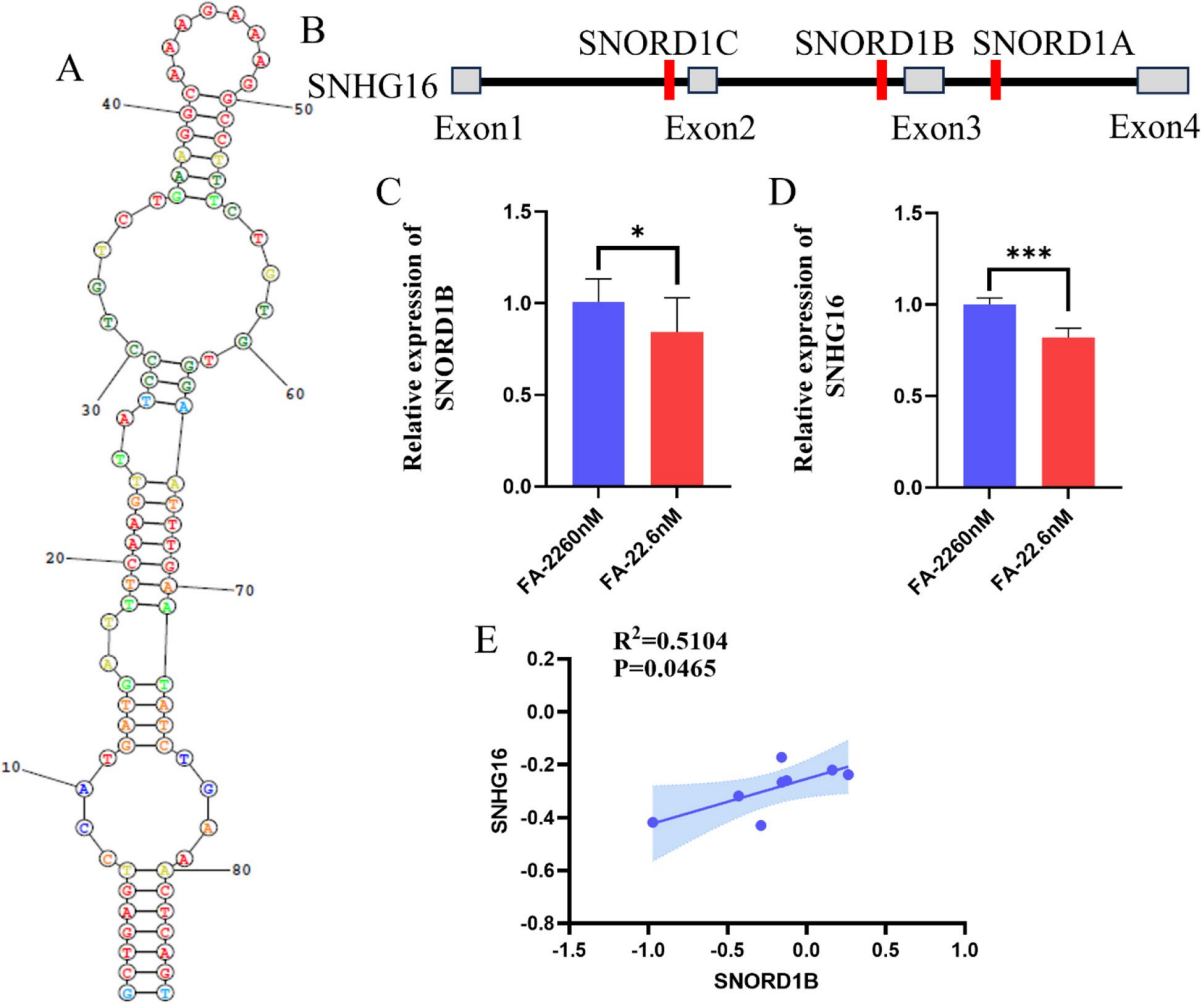
Effect of folate deficiency on *SNORD1B* and *SNHG16* expression. **A** Molecular structure of SNORD1B. **B** Gene structure of SNHG16. **C** Histogram of *SNORD1B* expression levels after 28 days of FA deficiency intervention. **D** Histogram of *SNHG16* expression levels after 28 days of FA deficiency intervention. **E** Correlation analysis of SNORD1B and SNHG16. All experiments were performed with three biological replicates (*n* = 3). compared with the control group, ^*^P < 0.05, ^**^P < 0.01, ^***^P < 0.001. The R^2^ value indicates the strength of the correlation

Through a comprehensive screening of 26 candidate snoRNAs, we identified SNORD1B as a functionally relevant candidate. This 86-nt C/D box snoRNA, which is hosted by the lncRNA SNHG16 gene, was selected due to its significant upregulation in HCC, its association with poor patient survival, and its involvement in folate-dependent 2'-O-ribose methylation. (Fig. 5A, B). However, its precise mechanistic role in liver cancer pathogenesis remains unclear. To investigate the relationship between folate availability and SNORD1B expression, we subjected HepG2 cells to a 28-day FA deficient intervention. This treatment resulted in a significant reduction in both SNORD1B and SNHG16 (Fig. 5C, D). Furthermore, we observed a strong positive correlation between their expression levels (R^2^ = 0.5104, *P* = 0.0465), suggesting coregulation (Fig. 5E). Collectively, these data indicate that FA deficiency transcriptionally represses the host gene SNHG16, leading to suppressed SNORD1B biogenesis.

### FA Deficiency Impairs 2′-O-Methylation of 28S rRNA and ribosome activity

**Fig. 6:**
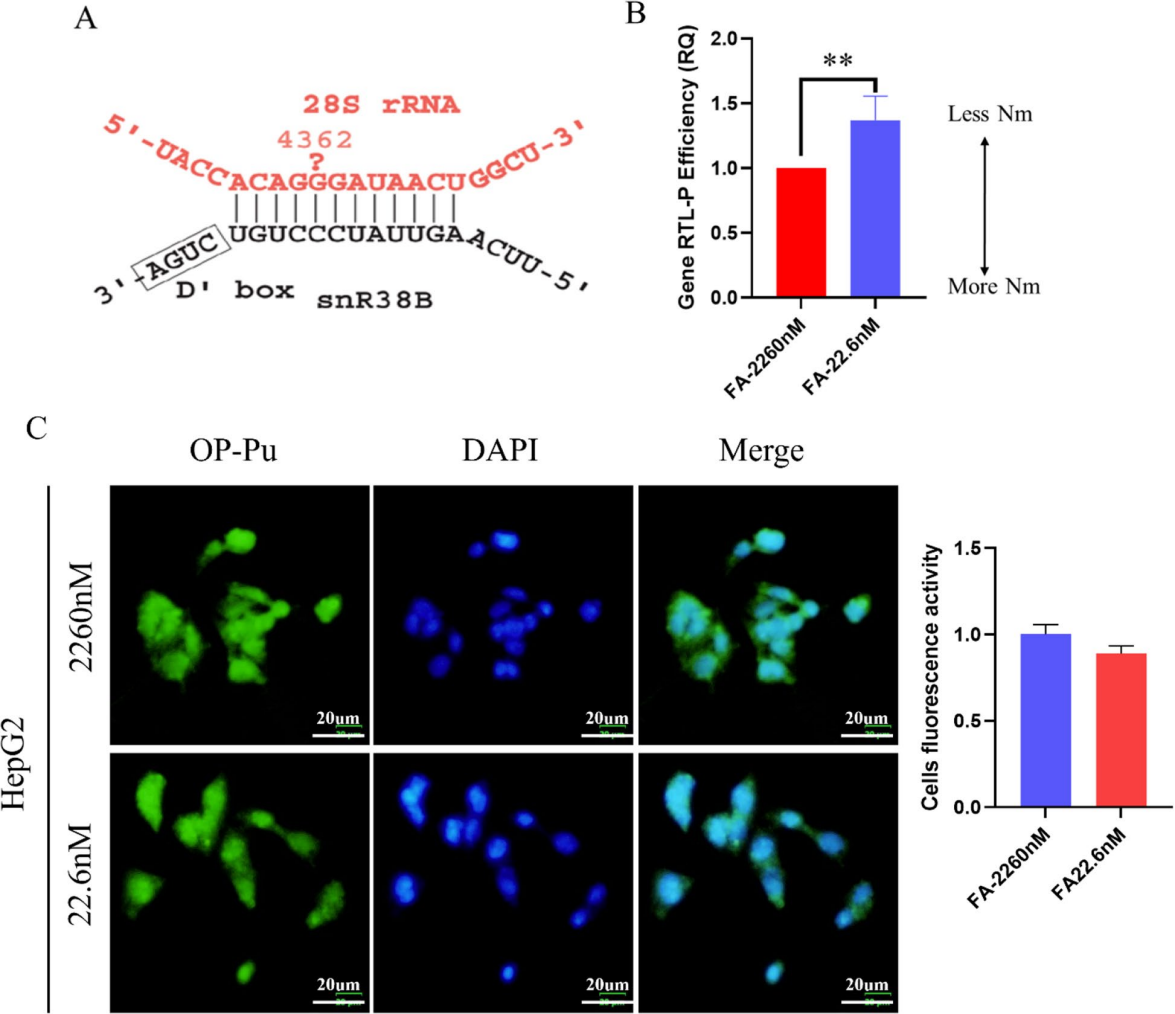
Folate deficiency impairs SNORD1B-dependent 2′-O-methylation and reduces ribosomal activity. **A** Predicted secondary structure of SNORD1B. **B** 2′-O-methylation levels at site G4362 on 28S rRNA after 28 days of folate deficiency. **C** Ribosomal activity assessed by OP-puromycin incorporation (green) in HepG2 cells following 28-day folate intervention. Nuclei were counterstained with DAPI (blue). Scale bar = 20 μm. All experiments were performed with three biological replicates (*n* = 3). ^*^P < 0.05, ^**^P < 0.01

Bioinformatic analysis identified G4362 on 28S rRNA as the predicted target site of SNORD1B (Fig. 6A). We observed that FA-deficiency HepG2 exhibited significantly reduced 2′-O-Me levels at 28S rRNA G4362 (Fig. 6B), demonstrating that FA-deficiency mediated SNORD1B downregulation impairs its methylation-guiding function. OP-Puro incorporation assays suggested a potential reduction in global protein synthesis in FA-deficient HepG2, although this trend did not reach statistical significance (Fig. 6D). Together, our findings define a mechanism whereby folate deficiency impairs ribosomal function by suppressing SNORD1B-dependent 2'-O-methylation at the G4362 site of 28S rRNA.

### FA deficiency alters snoRNP components, cell cycle, and inflammatory pathways

**Fig. 7:**
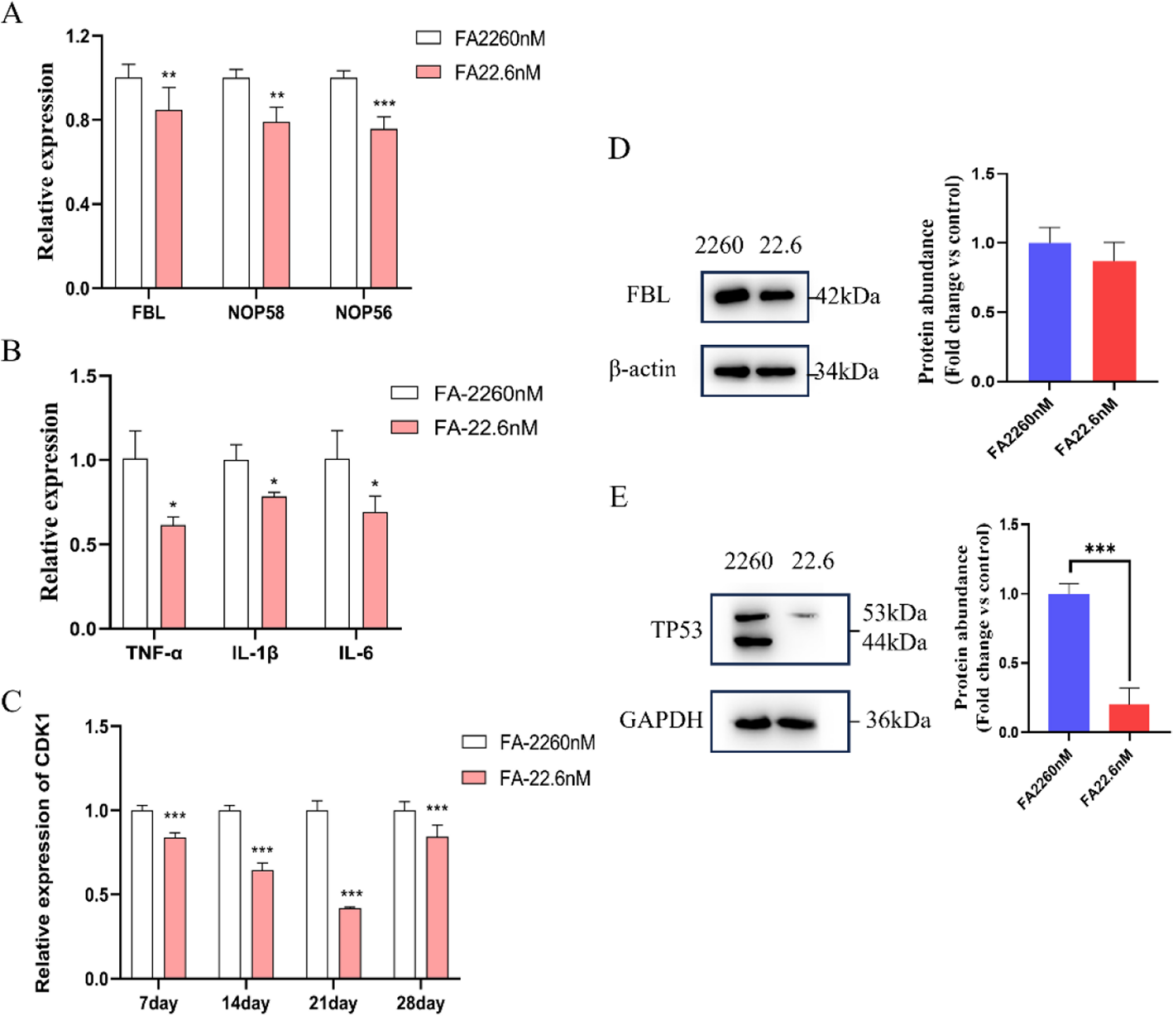
Effects of FA deficiency on the expression of snoRNA-binding proteins, inflammatory factors, and cell cycle-related factors in HepG2 cells. **A** mRNA levels of FBL, NOP58, and NOP56 after 28 days of intervention. **B** mRNA levels of TNF-α, IL-1β, and IL-6 after 28 days of intervention. **C** mRNA levels of CDK1 after different durations of intervention. **D** Protein expression level of FBL after 28 days of intervention. **E** Protein expression level of TP53 after 28 days of intervention. All experiments were performed with three biological replicates (*n* = 3). ^*^P < 0.05, ^**^P < 0.01, ^***^P < 0.001 vs. control group

FA deficiency significantly suppressed the mRNA levels of key snoRNP protein components (FBL, NOP58, and NOP56), potentially disrupting the complex's role in rRNA methylation (Fig. 7A), while FBL protein expression revealed a decreasing trend (Fig. 7D). Following 28 days of folate deficiency, a marked transcriptional suppression of the pro-inflammatory cytokines TNF-α, IL-1β, and IL-6 was observed (Fig. 7B). Based on the predicted functional link between SNORD1B and TP53 (RNALnter, confidence score = 0.5831; Fig. S3) and prior evidence of TP53-mediated rRNA suppression, we hypothesized an impact on cell cycle regulation. Consistent with this, CDK1 expression was significantly suppressed across all time points of FA deficiency (Fig. 7C). Furthermore, there is a pronounced reduction in TP53 protein levels after 28 days (Fig. 7E). In summary, folate deficiency orchestrates a broad cellular response by disrupting snoRNP biogenesis through coordinated downregulation of its core components, which in turn impairs rRNA methylation and ultimately suppresses both cell-cycle progression and pro-inflammatory signaling in HepG2.

### FA deficiency inhibits the proliferation and migration of HepG2 cells

**Fig. 8:**
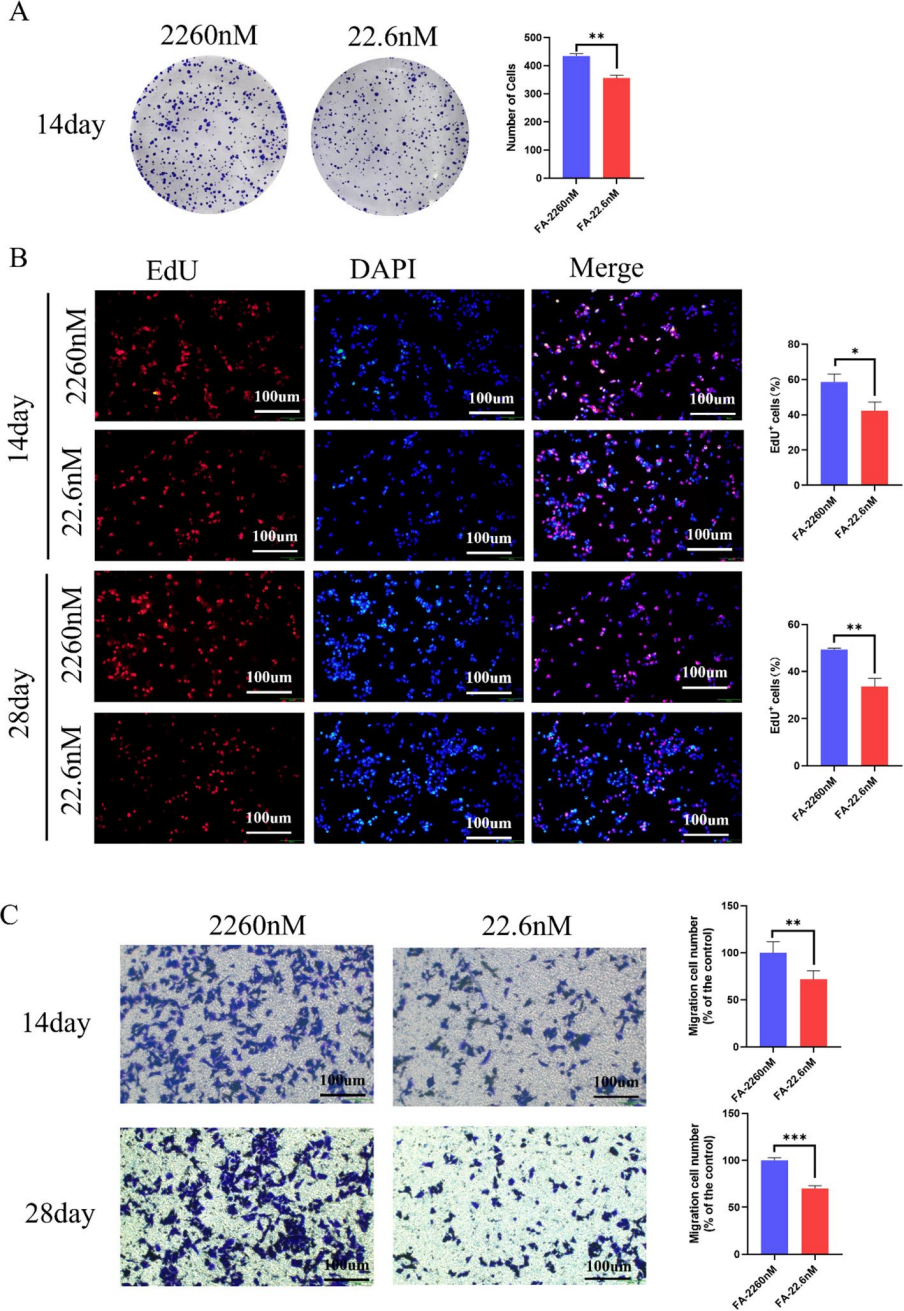
Effect of FA deficiency intervention on HepG2 cell proliferation and migration. **A** Results of plate clone formation assay for cell colony formation ability. **B** The EdU assays were also performed to measure the proliferation. Scale bars: 100 µm. **C** Transwell assays were performed to evaluate cell migration ability. Scale bars: 100 µm. All experiments were performed with three biological replicates (*n* = 3). ^*^P < 0.05, ^**^P < 0.01, ^***^P < 0.001; nM: nmol/L

Functional enrichment analysis of the differentially expressed snoRNAs indicated their primary association with genes and pathways involved in cell proliferation. To validate this finding, we assessed the proliferative and migratory capacities of HepG2 cells following 14 and 28 days of FA deficiency. At the 14-day, the plate clone formation assay showed that FA deficiency significantly attenuated the proliferative ability of HepG2 cells (Fig. 8A). This result was corroborated by EdU staining, which also indicated a clear inhibitory effect (Fig. 8B). By day 28 of FA deficiency, the anti-proliferative effect became more pronounced, as evidenced by a further reduction in EdU incorporation (Fig. 8B). Similarly, transwell migration assays revealed that FA deficiency significantly suppressed cell migration at 14 days, with this inhibitory effect being substantially stronger after 28 days (Fig. 8C).

## Discussions

Folic acid is an essential nutrient that supports nucleotide synthesis and methylation. Its role in cancer is defined by a dual role: protecting against tumor initiation while potentially accelerating the advancement of established cancers [30, 31]. Resolving this paradox is critical for understanding its precise impact on tumor biology. SnoRNAs, a class of non-coding RNAs, are garnering increasing interest for their association with HCC and their detectable presence in bodily fluids, underscoring their promise as clinical biomarkers [24, 32, 33]. While snoRNAs are typically intron-encoded and co-regulated with their host genes [34], emerging evidence suggests that certain snoRNAs can become uncoupled from host gene transcription, either by achieving independent expression or by reciprocally modulating the splicing of their host transcript. One major pathway facilitating this uncoupling is nonsense-mediated mRNA decay (NMD), which selectively degrades the host transcript but preserves the intronic snoRNA [35]. Strikingly, only about 60% of snoRNAs correlate positively with their host gene levels, with the remainder showing no or negative correlation, evidencing a highly heterogeneous regulatory regime [36].

Studies have established that DNA methylation suppresses snoRNA biogenesis, serving as a key mechanism of epigenetic silencing in human cancers. For instance, the 5′-CpG islands of host genes for snoRNAs including SNORD123, U70C, and ACA59B are frequently hypermethylated in cancer cells but remain unmethylated in normal tissues. This hypermethylation is closely linked to transcriptional repression of the associated snoRNAs, a phenomenon prevalent across multiple cancer types, particularly in leukemia [37]. Another study demonstrated the tumor-suppressive role of SNORD113-1 in HCC, revealing significant promoter hypermethylation at its host gene in tumor tissues compared to adjacent non-tumor samples [25]. These findings consolidate the model in which CpG methylation at snoRNA host gene promoters directly governs snoRNA silencing in cancer. Since folate (as 5-methyltetrahydrofolate) provides the methyl groups for DNMT-mediated DNA methylation [38], we propose that FA deficiency indirectly regulates snoRNA expression by altering the methylation profiles of their host gene promoters.

Our study revealed that folate deficiency markedly reshapes the snoRNA expression profile, with 26 differentially expressed snoRNAs (12 upregulated and 14 downregulated). The downregulated snoRNAs were associated with the control of epithelial and muscle cell proliferation and organ morphogenesis, mediated primarily by perturbations in growth factor, second messenger, and interleukin signaling pathways. These observations affirm a key role for these snoRNAs in governing cell proliferation and suggest their involvement in development and tissue formation, thereby connecting snoRNA-mediated rRNA modification to fundamental biological processes [39]. KEGG analysis identified HCC as the most significantly enriched disease, corroborating a direct link between FA-responsive snoRNAs and hepatocarcinogenesis. Subsequent protein–protein interaction analysis pinpointed central hub proteins, including TP53, CDK, and EGFR. Furthermore, significant enrichment of NRTK and PI3K pathways further implicated these snoRNAs in proliferation and oncogenic processes [40]. Collectively, these results establish snoRNAs as critical mediators of malignant transformation.

Analysis of TCGA data identified 14 snoRNAs with differential expression in HCC. Of these, 13 were up-regulated (suggesting potential oncogenic roles), while SNORD73B was down-regulated (implying tumor-suppressive functions). The distinct expression patterns of these snoRNAs indicate their functional relevance in HCC pathogenesis. Furthermore, survival analysis associated high expression of SNORA36C and SNORD1B with poor patient prognosis. Collectively, our findings suggest that FA deficiency modulates snoRNA expression to influence proliferative pathways, highlighting the potential of these snoRNAs as diagnostic biomarkers and therapeutic targets in HCC.

We selected SNORD1B for mechanistic investigation and found that folate (FA) deficiency downregulated both SNORD1B and its host gene SNHG16. This downregulation consequently reduced 2′-O-ribose methylation at site G4362 of the 28S rRNA, thereby impairing ribosomal function. Furthermore, following 28 days of FA deficiency, the transcript levels of three core C/D box snoRNA-binding proteins were significantly suppressed. A concurrent decreasing trend was also observed for the FBL protein, which is typically overexpressed in hepatocellular carcinoma and is a known target of TP53-mediated suppression. Folate deficiency downregulates CDK1 transcription and TP53 protein in HepG2 [41, 42]. Considering the pivotal functions of TP53 in arresting the cell cycle via p21 activation, initiating apoptosis, and blocking angiogenesis [43–45], the observed alterations in snoRNA expression and downregulation of these critical regulators point to a coordinated mechanism through which folate deficiency ultimately disrupts cell cycle progression.

Our analysis positioned the downregulated snoRNAs, including SNORD1B, within interleukin-signaling pathways, thereby linking them to the pro-tumorigenic inflammatory environment of HCC. This is highly relevant as key cytokines in these pathways, such as TNF-α, IL-1β, and IL-6, are known drivers of chronic liver inflammation and subsequent HCC development [46, 47]. Our results demonstrate that FA deficiency significantly downregulated TNF-α, IL-1β, and IL-6 expression and, when maintained at 22.6 nM for 14–28 days, progressively inhibited the proliferation and migration of HepG2. This suggests that FA restriction may suppress HCC development by disrupting snoRNA-mediated ribosomal function and protein synthesis, thereby suppressing tumor-promoting pathways.

As a micronutrient obtainable solely through diet, folate presents a unique therapeutic advantage: its restriction can modulate cancer growth without disrupting cellular energy supply, unlike macronutrient interventions. This positions FA-level targeting as a precise strategy in cancer therapy [48]. While the anti-proliferative effect of folate deprivation has been largely attributed to impaired nucleotide and amino acid biosynthesis leading to cell cycle arrest, our study reveals a novel, complementary mechanism. We demonstrate that FA deficiency alters host gene methylation, which in turn dysregulates snoRNA expression and compromises site-specific rRNA methylation. This cascade ultimately suppresses ribosomal activity and global protein synthesis, representing a previously unrecognized pathway through which FA restriction inhibits cell proliferation. Our findings substantially expand the mechanistic understanding of folate in cancer biology and open new avenues for therapeutic and preventive strategies.

## Supplementary Information

Supplementary Material 1.

## Data Availability

All the data generated or analyzed during this study are included in this published article. The database used in the current study is TCGA (https://portal.gdc.cancer.gov/).
