# Supplementary material for: Folate-driven changes in snoRNA function: a novel epigenetic-ribosomal axis in hepatocellular carcinoma
Source: Hereditas. 2026 Mar 23;163:56. doi: 10.1186/s41065-026-00666-x (PMC13130495; doi:10.1186/s41065-026-00666-x)
Supplement: Supplementary file 1 — Supplementary Material 1. [file 41065_2026_666_MOESM1_ESM.docx]

**Supplementary materials**

**Table S1** List of primers sequences used in this study

| Names | Primer sequences (5′-3′) |
| --- | --- |
| GAPDH-F | AGATCATCAGCAATGCCTCCT |
| GAPDH-R | GGTCATGAGTCCTTCCACGA |
| SNORD11-F | TACAACACACCCAACAGGAATCT |
| SNORD11-R | GTTCATAGGGAAGCCACAAGTTT |
| SNORD50A-F | GGATCCTATCTGTGATGATCTTATCC |
| SNORD50A-R | GAGCTCATCTCAGAAGCCAGATCCGT |
| SNORA38B-F | TTCCTTGTCTTTGGACATGTAAGA |
| SNORA38B-R | TGTGGGATGGTTGATCTTGA |
| SNORD58A-F | GCAGTGATGACTTTCTTAGGAC |
| SNORD58A-R | GCTGCTCAGAATTTATTAATTTTCACGGT |
| SNORA70F-F | CTGCAGTCAATTAAGTGTACTG |
| SNORA70F-R | CCCCATAAGGAAAGGAAC |
| SNORA12-F | AGTTGTGGTGGTTTTCTTTTT |
| SNORA12-R | CTTTGCCATCTCCGATATTAT |
| SNORA73B-F | ACAGTCCCTTTCCACAACG |
| SNORA73B-R | AGGCCCAGCTTCATCTTC |
| SNORA36C-F | GAGATGATTGGGTAGAAAGC |
| SNORA36C-R | GGGAGGCAAATGAATAAAA |
| SNORD16-F | TGTTCTCAGCGACAGTTG |
| SNORD16-R | ATTTTCGTCAACCTTCTG |
| SNORD62A-F | TCCTTCTGACCCTCCACT |
| SNORD62A-R | TGTCCTCTTCATCTCCCT |
| RNU105B-F | ATACACCCAGGAGGTCACTCT |
| RNU105B-R | CCCTATGCTACTTGGACACAG |
| SNORD101-F | TGACTTTAATTGTCGGATAC |
| SNORD101-R | AAAGGAGTGAAGGGGTAT |
| SNORD1B-F | TGATGATTTCAAGTTATCCCTGTC |
| SNORD1B-R | TTTCAGATATTCAAATTCCACACA |
| SCARNA14-F | AAACAGATGGTTCCTGCCGACA |
| SCARNA14-R | TGGAGCCATCGCACAGAAAATC |
| SNORD80-F | GCAGACTAACGCTGATGA |
| SNORD80-R | CAGATAGGAGCGAAAGAC |
| SNORD49A-F | ACTAATAGGAAGTGCCGTCA |
| SNORD49A-R | GTCTTCGTCAGTTATCGCT |
| SNORD18A-F | ACTTCATTGGTCCGTGTTT |
| SNORD18A-R | CAGAACATCCGAGAAAATC |
| SNORD59A-F | AAAATGACTTTCGTTCTTC |
| SNORD59A-R | CTCAGTACCTAAATGTGGC |
| SNORA67-F | AAGTAGCAGCTTGGAATAGAA |
| SNORA67-R | AAGGAAGGCAGAGGAAATA |
| SNORD41-F | TGTGACTGTTGATGTGGAA |
| SNORD41-R | GTACGAATACGCGATAAAT |
| SNORD1A-F | CACAAGCCTATGATGGTTA |
| SNORD1A-R | AGATTATTTCCCTCAGTCC |
| SCARNA5-F | GAATGTCACGGTCCCTT |
| SCARNA5-R | GAATCCCAAATTATCAGC |
| SNORD51-F | ATCTTTCGGCTGAGTTCG |
| SNORD51-R | AAAAGCAAATCCATCACG |
| SNORD103B-F | ACCCACTTGCCCTCACTG |
| SNORD103B-R | CAAAGATTCTCATTACCG |
| SNORA71C-F | CATTGGTAGTGCAGGGAGA |
| SNORA71C-R | AGGGTTTGGAAGAGACAGG |
| SNORA70E-F | GCCGACCTAGTTCCTTTC |
| SNORA70E-R | TCCTTTAGAGCAACCCATAC |
| SNHG16-F | GCAGAATGCCATGGTTTCCC |
| SNHG16-R | GGACAGCTGGCAAGAGACTT |
| CDK1-F | AAACTACAGGTCAAGTGGTAGCC |
| CDK1-R | TCCTGCATAAGCACATCCTGA |
| SNHG16-F | GCAGAATGCCATGGTTTCCC |
| SNHG16-R | GGACAGCTGGCAAGAGACTT |
| FBL-F | CCAAGAAGAGGACCAACATCAT |
| FBL-R | GAGGCTGTGGAGTCAATGC |
| NOP58-F | GGCAGCGTGTTCTGATTCTT |
| NOP58-R | GCCTGCCAGAAGCTGTAGAGT |
| NOP56-F | CTGGAGGAGCTGACAATGGA |
| NOP56-R | CACCACACGACTGGAGAAGC |
| 28S G4362-F | TGGGTTTTAAGCAGGAGGTGT |
| 28S G4362-R | GACGTCGCTATGAACGCTTG |


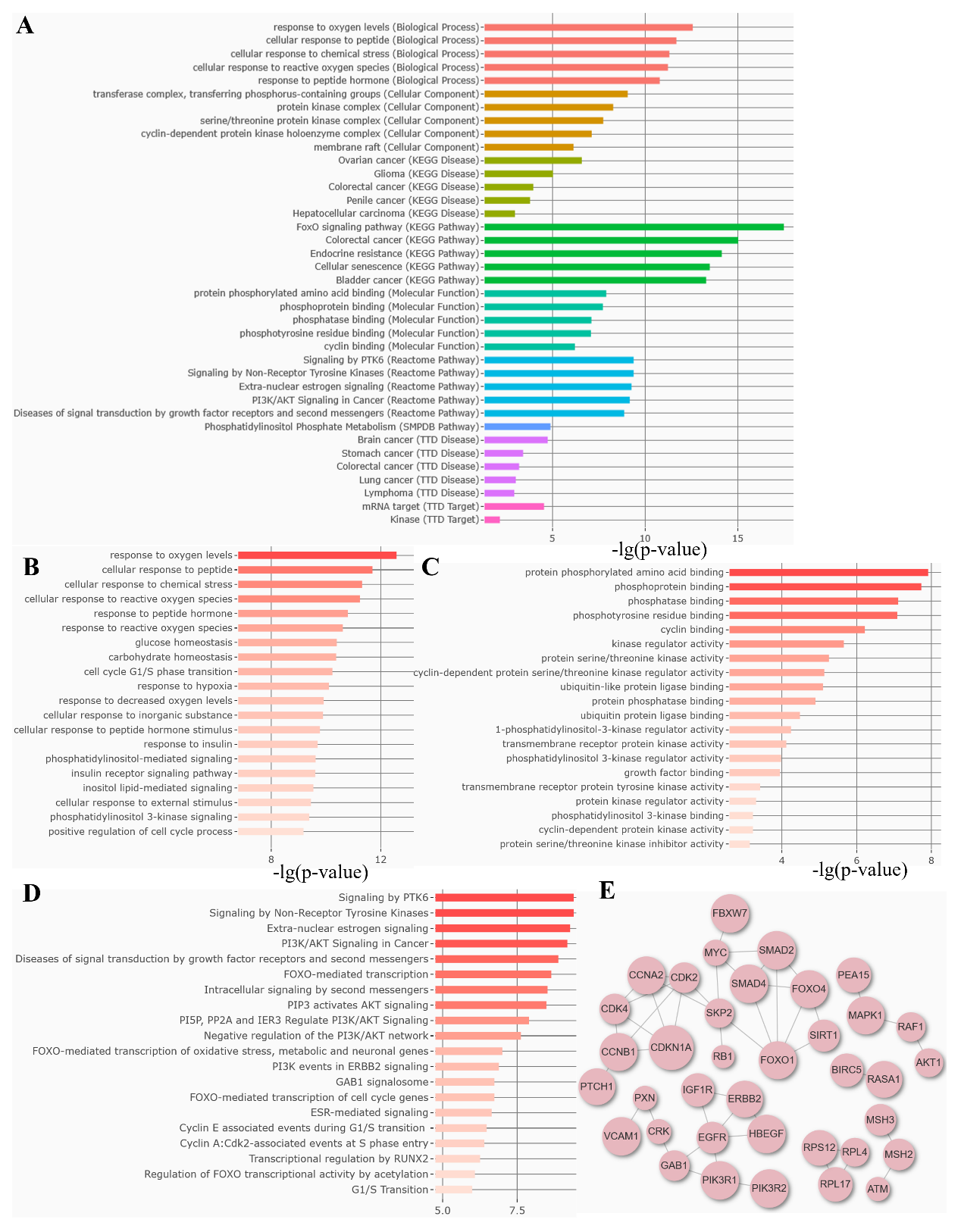


**Figure S1. Gene set enrichment analysis of 12 upregulated snoRNAs.** (A) Database summary. (B) Biological process enrichment. (C) Molecular function enrichment. (D) Reactome Pathway enrichment. (E) Protein interaction network. Nodes and edges represent the relationships and the strength of interactions between them.


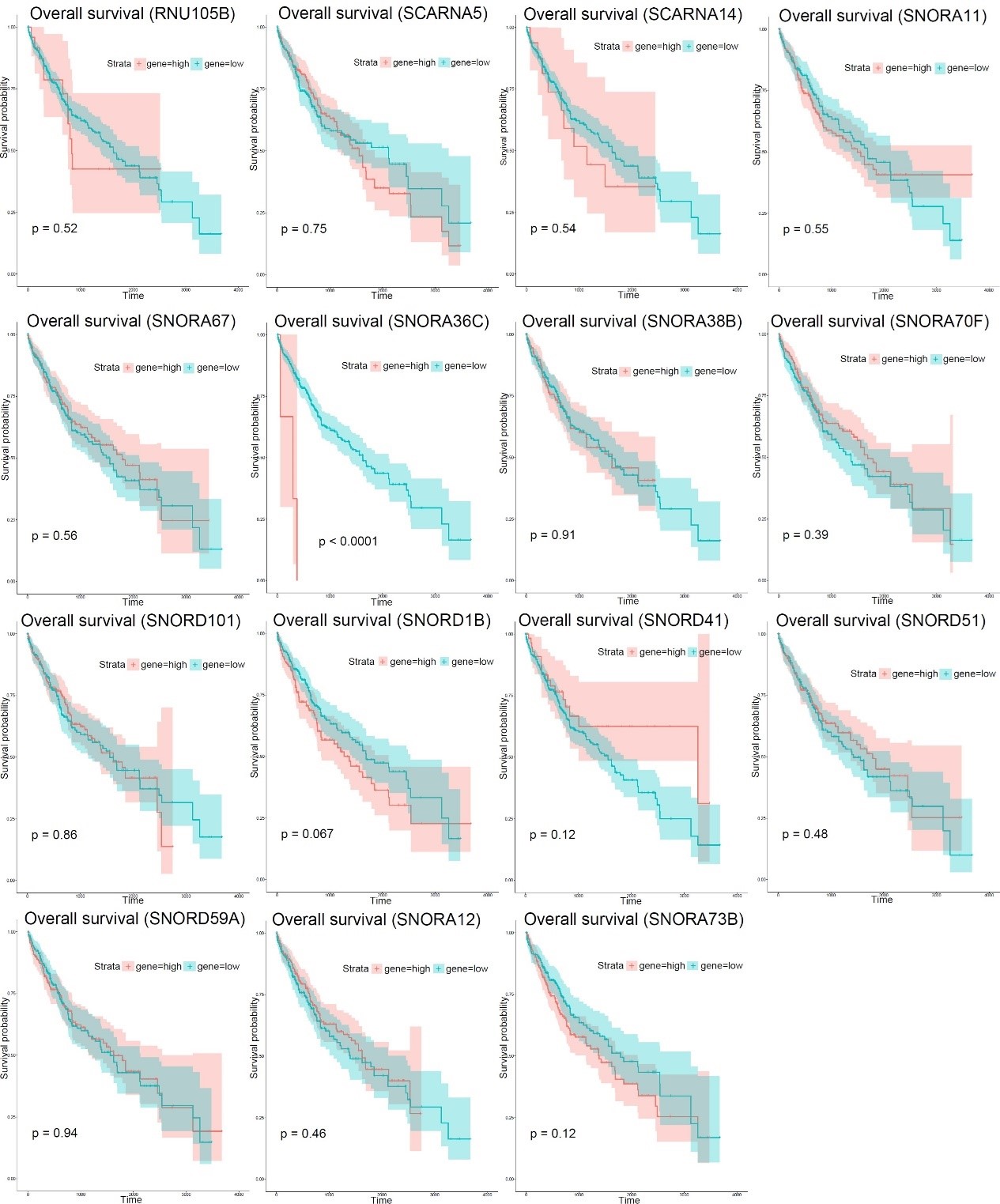


**Figure S2. Kaplan-Meier (K-M) survival analysis of liver cancer patients was performed based on the low and** **high expression of snoRNA by dichotomy.** The red and blue lines respectively represent high expression samples and low expression samples.


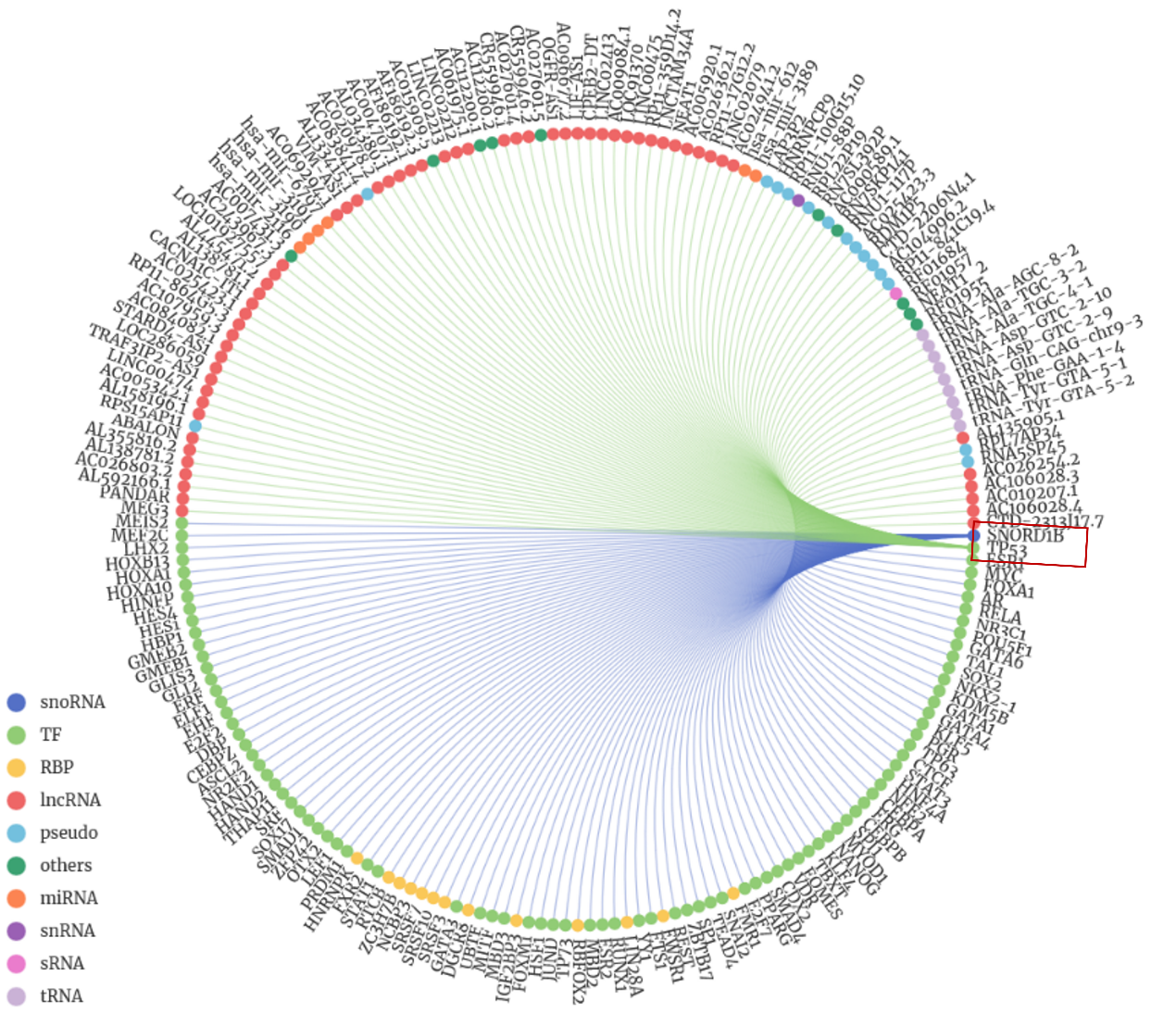


**Figure S3. RNAInter-predicted interaction network between SNORD1B and TP53.**
